# Discovering Implied Serial Order Through Model-Free and Model-Based Learning

**DOI:** 10.3389/fnins.2019.00878

**Published:** 2019-08-20

**Authors:** Greg Jensen, Herbert S. Terrace, Vincent P. Ferrera

**Affiliations:** ^1^Department of Psychology, Columbia University, New York, NY, United States; ^2^Department of Neuroscience, Columbia University, New York, NY, United States; ^3^Department of Psychiatry, Columbia University, New York, NY, United States

**Keywords:** reinforcement learning, model-free learning, model-based learning, cognitive maps, transitive inference

## Abstract

Humans and animals can learn to order a list of items without relying on explicit spatial or temporal cues. To do so, they appear to make use of transitivity, a property of all ordered sets. Here, we summarize relevant research on the transitive inference (TI) paradigm and its relationship to learning the underlying order of an arbitrary set of items. We compare six computational models of TI performance, three of which are model-free (*Q*-learning, Value Transfer, and REMERGE) and three of which are model-based (RL-Elo, Sequential Monte Carlo, and Betasort). Our goal is to assess the ability of these models to produce empirically observed features of TI behavior. Model-based approaches perform better under a wider range of scenarios, but no single model explains the full scope of behaviors reported in the TI literature.

## Introduction

Transitivity is a property of all ordered sets, including number systems, social hierarchies, rational economic preferences, and spatial position. Exploiting transitivity can reduce task complexity when learning serial order, since knowing that X > Y and Y > Z is sufficient to infer that X > Z. Using this implied information is called transitive inference (TI) and is thought to underlie some forms of serial learning. In this review, we describe and implement six reinforcement learning algorithms for serial learning. Three are model-free, and depend on expected reward value to make their judgments. The other three are model-based, and represent the rank associated with each stimulus using a spatial continuum. These algorithms are compared side-by-side using simulation under identical experimental conditions. The performance of the algorithms illustrates the utility of one-dimensional cognitive maps for representing abstract relationships.

Serial learning is ubiquitous, but the mechanisms underlying TI remain poorly understood. In humans, reasoning about serial order or rank has been studied for over 100 years, with a central role in the history of intelligence testing (Burt, 1911) and child development (Piaget, 1921). Following the demonstration that squirrel monkeys can perform TI (McGonigle and Chalmers, 1977), a wealth of results with animal subjects has revealed the breadth of serial learning. To date, every vertebrate species tested has shown evidence for some form of TI (Vasconcelos, 2008; Jensen, 2017). Given this broad comparative literature showing serial learning under various experimental procedures (Terrace, 2012), it seems reasonable that these abilities reflect cognitive mechanisms that have deep evolutionary roots.

Despite the ubiquity of this phenomenon, no consensus has emerged about a mechanism for inferring serial order. Some explain serial learning in animals using model-free learning (e.g., Vasconcelos, 2008). In the context of TI, we use the term “model-free learning” to refer to an algorithm that estimates a probability or rate of some outcome, conditional only on past observed events and on cues discernable in the environment. For example, a subject might learn that *p*(food) is low because food is rarely available in its past experience, but also learn that *p*(food | buzzer) is high because food is often made available while the buzzer is audible. Given sufficient training, even naïve conditional probability estimation of this kind can solve a wide variety of tasks without any deeper understanding of causal relationships.

Another approach for explaining behavior consistent with TI is “model-based learning,” in which observed events are presumed to reflect some underlying set of rules or relations. Subjects make additional assumptions about what the stimuli mean and how they relate to one another, and these assumptions support more complex inferences. Although cognitive models of serial learning often follow the themes of model-based learning, few are described in sufficient detail to simulate behavior, making it difficult to compare cognitive models to their model-free counterparts (Gazes et al., 2012). For serial learning, we believe that the most promising approach is to assume that each item in the list has some “spatial position” along an abstract continuum (Jensen et al., 2013, 2015).

Solving TI using a spatial representation is akin to an abstract cognitive map (Redish, 1999), an organizational scheme that could be used for much more than spatial navigation (Behrens et al., 2018). According to this proposal, positions of stimuli are “mapped” along a single dimension, a capacity that has been attributed to hippocampal computation (Eichenbaum et al., 2016). Recent studies on the computational capabilities of circuits in hippocampus (Oliva et al., 2016) and entorhinal cortex (Bellmund et al., 2016) further suggest that spatial modeling is a common substrate across many different cognitive domains (Constantinescu et al., 2016).

Viewed as spatial computations, TI problems become an important limiting case. In open fields (Chalmers et al., 2016) or radial arm mazes (Ferguson et al., 2019), the success of reinforcement learning often depends on physical and temporal cues. These allow a model-free algorithm to “navigate a space” without encoding a representation of that space, because the cues provide many ways to discover rewards. For example, early difficulties experienced by model-free learning of the 1983 Atari game *Ms. Pac-Man* (e.g., Mnih et al., 2015) have largely been overcome by making better use of the information on screen (e.g., van Seijen et al., 2017). In effect, the environment bears the burden of representing itself, freeing the agent from having to do so using an encoding scheme.

Algorithms of this kind struggle when task information is sparse. The 1984 Atari game *Montezuma’s Revenge*, for example, remains largely unsolved by reinforcement learning because it provides the user with very few informative cues (Justensen et al., 2019). Similarly, TI tasks present the bare minimum of information that only implies the list order, while other cues (such as time, stimulus position, and trial order) are carefully controlled to provide no information usable by the subject. As a result, TI procedures eliminate the cues that make open field problems with spatial landmarks solvable by model-free methods. TI tasks thus have the potential to reveal clues about the machinery that organisms use for spatial cognition in general, beyond the narrow scope of ordered lists.

In this review, we will first characterize the problem posed by TI tasks (see Section Problem: How to Infer Serial Ordering from Pairwise Comparisons), then describe three model-free learning algorithms (see Section Model-Free Solutions: Expected Value Estimation) and three model-based learning algorithms (see Section Model-Based Solutions: Inference About Ordering) that have been suggested as models of this kind of inference. We then recover the relevant algorithm parameters from empirical data and use them to perform simulations to evaluate how well these algorithms do under a variety of experimental conditions (see Section Simulation of Serial Order Tasks). We conclude with a brief model comparison analysis (see Section Model Comparison and Ensemble Modeling), a note on computational complexity (see Section A Note on Computational Complexity), and our concluding thoughts (see Section Conclusion).

## Problem: How to Infer Serial Ordering From Pairwise Comparisons

When organisms solve the TI problem, they in effect perform a statistical procedure: Inferring ranks of items in an ordered list, based on partial information provided by a series of pairwise comparisons. Such statistical inferences can be accomplished with a variety of strategies (Bürkner and Vuorre, 2018). Because many candidate algorithms exist, above-chance performance in a TI task is not sufficient to determine how organisms solve the task. Instead, common features of behavior that accompany this inference provide vital clues about its underlying computations.

### Experimental Evidence

**FIGURE 1:**
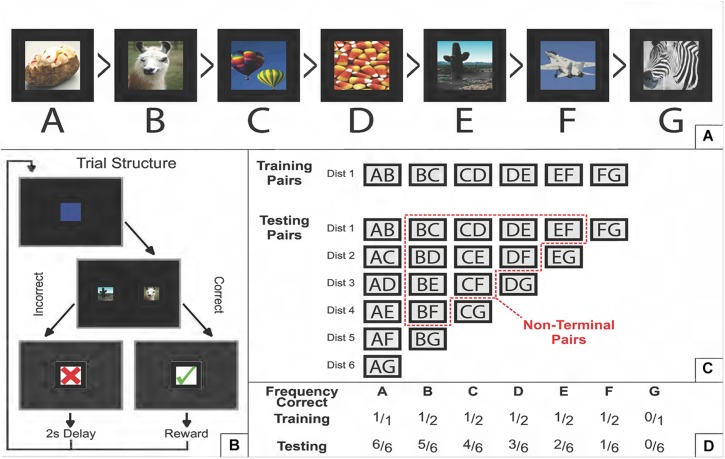
Schematic description of a transitive inference task. **(A)** Example of a 7-item ordered list of photographic stimuli. “Greater Than” symbols denote which items dominate which other items (e.g., baked potato is correct relative to every other stimulus, but fighter plane is only correct when paired with zebra). **(B)** Example of a single trial. Following a start stimulus, two stimuli appear on screen. If the correct item is selected, positive feedback is provided; an incorrect choice leads to negative feedback. In every trial, there is always one correct and one incorrect answer. No more than two stimuli are ever presented at a time for the duration of the experiment. **(C)** The training and testing sets of pairs of stimuli. During training, only adjacent pairs (AB, BC, etc.) are presented typically in a randomized order that counterbalances how often each stimulus appears on each side of the screen. During testing, all 21 pairs are presented. The “symbolic distance” (denoted as “Dist”) refers to how many steps in the list one has to make to get from one item to another. Adjacent pairs have a symbolic distance of 1, whereas pairing both terminal items has a distance of 6. The “non-terminal pairs” are those that exclude both the first and last list item. If a non-terminal pair was also excluded from the training set, then it considered is a “critical pair” at test. **(D)** The frequency with which each stimulus is correct, relative to the number of pairs it appears in during that phase. During training, non-terminal items are correct 50% of the time, and consequently comparisons of the overall reward rate for non-terminal are equivocal, given only the information available during training. Reproduced with permission (DOI: 10.6084/m9.figshare.7992005.v1). Copyright 2019, Jensen, Terrace, and Ferrera.

In the standard TI task, subjects are presented with pairs of stimuli drawn at random from an ordered list, and are rewarded for selecting the “correct” item. A stimulus is correct if its rank is lower than the distractor with which it is paired. So, for example, given the 7-item ordered list ABCDEFG (e.g., Figure 1A), the stimulus C is a correct answer in the context of the pair CD, but is incorrect in the context of the pair BC (Figure 1B).

Although there are 21 possible pairs in a 7-item list, training all the pairs in parallel is not sufficient to conclude that a “transitive inference” has occurred. A model-free learning mechanism that tracks the expected value of each stimulus can succeed in an all-pairs design, because A is correct in more pairs than B, which in turn is correct in more pairs than C, and so forth. To provide a strong test that an organism’s inference of list order depends on transitivity (rather than comparing each item’s association with obtained rewards), the classic test of TI is to train *only* the adjacent pairs (in this case, AB, BC, CD, DE, EF, and FG). This contrast between training sets and testing sets is shown in Figure 1C. Under adjacent-pair training, terminal items A and G are associated with perfect information (100 and 0% rewards, respectively), while all non-terminal items are correct in 50% of the training cases (Figure 1D). Upon completion of training, B and D have identical expected values, providing no clue about which response is correct. We henceforth refer to pairs that are non-adjacent (i.e., outside the training set) and non-terminal (i.e., ambiguous with respect to expected value) to be the *critical pairs*. If a subject exceeds chance on those pairs, some inference beyond mere reward associations must have taken place. In a 7-item list, the six critical pairs are BD, CE, DF, BE, CF, and BF.

Aside from above-chance performance on the critical pairs, two other major clues are routinely evident in studies of TI. The first of these is the *terminal item effect* (Wynne, 1997; Acuna et al., 2002), which occurs when pairs that include terminal items yield more correct responses. Since terminal items have an unambiguous reward history (e.g., A is always correct), these discriminations should be easier because of their differential reward associations, even if a non-associative mechanism is also at work. Terminal item effects are usually visible throughout training and testing. A model purporting to explain how subjects perform TI should display these effects.

The second consistent behavioral phenomenon is the *symbolic distance effect* (D’Amato and Colombo, 1990), which is present when the difference in the ranks of two items (or the pair’s “symbolic distance”) is associated with response accuracy. Non-human studies of TI almost always display lower accuracy for pairs of adjacent items than for pairs that are spaced apart more widely in the list (Jensen, 2017), even when adjacent pairs are extensively trained. Unlike terminal item effects (which can arise from reward associations), symbolic distance effects provide compelling evidence of a cognitive strategy (Terrace, 2010; Jensen et al., 2013), particularly when those distance effects can be seen among critical pairs that did not appear in the training set (e.g., Jensen et al., 2017; Kao et al., 2018).

One of the surprising predictions of a positive symbolic distance effect is that response accuracy should be lower for adjacent pairs than for any other distance, despite extensive training on those pairs. In non-human animals, even when training consists of tens of thousands of presentations of a small set of adjacent pairs (e.g., Merritt et al., 2007; Lazareva and Wasserman, 2012), those pairs nevertheless have the highest error rates. In some cases, response accuracies appear barely above chance throughout training, only to leap to higher accuracies as soon as pairs with larger symbolic distances are tested (e.g., Jensen et al., 2015). These results, particularly the high starting accuracy for non-adjacent pairs, suggests a representation that approximates the overall order successfully but retains uncertainty about the positions of individual items.

**FIGURE 2:**
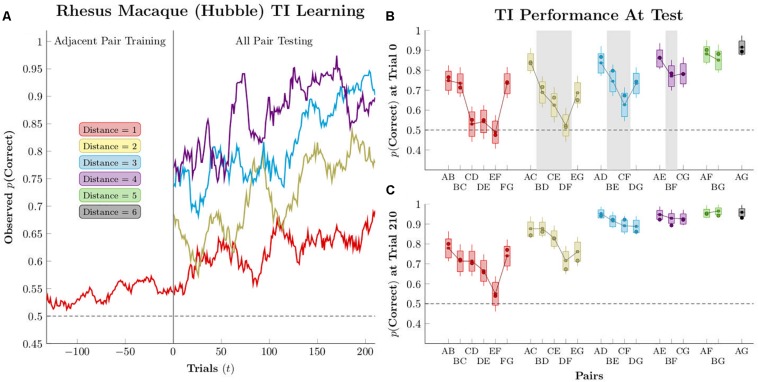
Estimated performance for a single subject (Hubble), as reported by Jensen et al. (2015). Circles represent empirical averages of observed performance, while box-and-whisker plots represent the estimated performance according to pairwise logistic regression. Boxes represent the 80% credible interval, whereas whiskers represent the 95% credible interval. **(A)** Mean response accuracy for non-terminal pairs of distances 1–4, smoothed over a 21-response moving window **(B)** Estimated performance at the start of testing (“trial zero”). Pairs shaded with gray are the “critical pairs” that are expected to remain at chance if subjects are engaged only in a reward association learning strategy; if their performance is above chance, it suggests that an actual inference is being performed. **(C)** Estimated performance after 210 trials of testing. Reproduced with permission (DOI: 10.6084/m9.figshare.7992008.v1). Copyright 2019, Jensen, Terrace, and Ferrera.

Figure 2 gives an example of typical TI performance (from Jensen et al., 2015). This monkey (“Hubble”) was given 150 to 300 trials of training on adjacent pairs (red points) in 7-item lists. Response accuracies were fit for each pair independently using logistic regression in the Stan programing language (Carpenter et al., 2017), as described in the Supplementary Material. Using the models for each of the pairs, Figure 2 projects the accuracy on the first trial of testing (“trial 0”), when each non-adjacent pair had not been seen before, as well as the last trial of testing (“trial 210”), at which point all pairs were familiar. The critical pairs (highlighted in gray) are above chance in five out of six cases at trial 0, and pairs with a larger symbolic distance (e.g., BF) tend to display higher performance than those with smaller symbolic distances (e.g., CE).

## Model-Free Solutions: Expected Value Estimation

Model-free approaches seek to explain TI (and by extension, serial learning) purely in terms of observable associative factors, making no reference to internal states or to representations of ordering (Wynne, 1995; Vasconcelos, 2008). These theories assume that model-free learning can account for above-chance performance on critical pairs. In studies of this kind, the phrase “transitive inference” is used to identify the behavioral phenomenon of above-chance performance on critical pairs, not to imply a logical inference *per se.*

Most models of this kind choose stimuli on the basis of the “associative strength” each has with rewards. That is, model-free learning relies exclusively on using feedback from the environment to evaluate the relative frequency of reward as a function of past experience. Experienced value is taken to predict future expected value, and choices should thus be made to maximize future rewards.

Acting to maximize rewards can be deceptively powerful, even in the absence of a representational (i.e., model-based) framework for understanding the task at hand. As such, we must be cautious about interpreting results that are consistent with a cognitive account. In some cases, TI results can also be explained by model-free accounts. We consider three model-free algorithms below.

### *Q*-Learning: Expected Value Weighted by Recency

One of the simplest incarnations of model-free learning that is relevant to TI problems is *Q*-learning (Watkins and Dayan, 1992). As it is typically implemented, *Q*-learning uses both a retrospective and a prospective learning mechanism. The retrospective element maintains a running average of the outcomes for each of its responses, weighted to favor recent events according to an exponential function (Glimcher, 2011). This retrospective element is identical to the “associative strength” estimated by the Rescorla–Wagner model (Widrow and Hoff, 1960; Rescorla and Wagner, 1972), with roots in the ‘mathematical model of simple learning’ proposed by Bush and Mosteller (1951). Its prospective element uses a discounted projection of the future state to which the current choice leads. Although *Q*-learning cannot explain TI in a well-controlled experiment, it provides a useful baseline against which to compare other algorithms.

Under *Q*-learning, each behavioral alternative has a numerical “quality,” which corresponds to the value or utility expected from performing that behavior, contingent on the “state” the actor is in. Typically, the algorithm’s state is determined by environmental context, such as position in space or the presence of an informative cue. At a time *t*, the value associated with an action *a*_*t*_, given some contingent contextual state *s*_*t*_, is denoted by *Q*(*a*_*t*_|*s*_*t*_) (to be read as “the value of action *a*_*t*_, given the state *s*_*t*_”). When a choice is made, its consequence *c*_*t*_ is used to update the value of *Q* according to the following function:

(1) $$Q\,\left(a_{t}|s_{t}\right)\leftarrow Q\,\left(a_{t}|s_{t}\right)+\delta \,\left[c_{t}+\gamma \,\left(\max\left(Q\,\left(\text{all}\,a_{t+1}|s_{t+1}\right)\right)\right)-Q\,\left(a_{t}|s_{t}\right)\right]\alpha \in \left(0,1\right),\gamma \in \left(0,1\right)$$

The term *c*_*t*_ is sometimes called the “immediate reward,” but *c*_*t*_ may also take on a negative value in the event that the consequence is aversive.

*Q*-learning’s value updating function has two parameters. The ‘learning rate’ δ controls retrospective learning, and governs how quickly the actor forgets the old information encoded in *Q*(*a*_*t*_|*s*_*t*_) in favor of new information. When δ is 0.0, the actor cannot learn from feedback; when it is 1.0, it discards all past information every time it observes a new outcome. Giving δ a low value (e.g., below 0.2) results in the gradual progress typical of trial-and-error learning.

The ‘discount factor’ γ controls *Q*-learning’s prospective learning, and governs the influence that future states have on estimates of the value of the current response. The quantity *max*⁡(*Q*(all *a*_*t* + 1_|*s*_*t* + 1_)) refers to the value of the best projected response option, contingent on the actor’s state in the next time step, *s*_*t+1*_. This also contributes to the value of *Q* because a current choice might bring the actor *closer* to a desirable contextual state, even if that choice doesn’t yield an immediate reward (e.g., moving the agent closer to the exit of a maze in order to hasten the receipt of a reward upon exiting). If γ = 0.0, *Q*-learning is identical to Rescorla–Wagner-style associative learning:

(2) $$Q\,\left(a_{t}|s_{t}\right)\leftarrow Q\,\left(a_{t}|s_{t}\right)+\delta \,\left[c_{t}-Q\,\left(a_{t}|s_{t}\right)\right],\alpha \in \left(0,1\right)$$

Despite its extreme simplicity, *Q*-learning can solve many difficult problems if given sufficient training and a sufficiently complex matrix of states and actions. This is because, when an actor’s choices (e.g., “move forward”) influence its contextual state (“distance and direction of food”), the algorithm can discover not only which actions yield rewards, but can extrapolate backwards from those rewards to discover which *sequences* of behavior lead to rewards. However, *Q*-learning is at a particular disadvantage in the case of TI tasks because most experimental designs predetermine and randomize the presentation order of stimuli, such that the actor’s choice *a*_*t*_ has *no effect whatsoever* on the subsequent state *s*_*t+1*_. If, for example, a subject sees the stimulus pair AB on the first trial, its choice has no influence on which pair is presented on the second trial, or on which side of the screen each stimulus appears. Put another way, because each future state *s*_*t+1*_ does not depend in any way on *a*_*t*_, the value of *max*⁡(*Q*(all *a*_*t* + 1_|*s*_*t* + 1_)) in Equation 1 is entirely determined by the task structure, and not by the actor’s choices. TI is thus akin to a maze in which, after each choice, the experimenter teleports the subject to another part of the maze.

Using *Q*-learning to try to solve TI tasks also requires a decision by its designer about what constitutes a “state.” If, for example, we treat each unique stimulus pairing (e.g., “BC” or “CD”) as a unique state, then the actor will treat *Q*(C|BC) as being entirely unrelated to *Q*(C|CD). When presented with a stimulus pairing it has never previously observed (e.g., BD), such an actor would always begin at chance, with no possibility of generalizing. We are interested in an algorithm that *can* generalize (since this is the only way this algorithm has any hope of solving novel scenarios), we have coded our *Q-*learning implementation to ignore context. The result is that *Q* is a vector, with one value for each response alternative.

Finally, the designer must decide whether to update response alternatives that are *not* chosen. For example, if the actor is presented with BC and chooses B (yielding a reward), it should update *Q*(B) using *c*_*t*_ = 1.0, but should it *also* update *Q*(C) using *c*_*t*_ = 0.0? A *Q*-learning algorithm that only updates the chosen response is hereafter identified as “asymmetric.” Asymmetric learning is commonly assumed in the behavior analysis literature, because the tradition of behaviorism insists that behavior should only be explained by observables, not by counterfactuals. We call algorithms that update the values of both choices “symmetric,” since they exploit the either/or symmetry of the TI task, effectively extracting two trials worth of information from each trial. We feel this choice can be justified on the grounds that subjects familiar with the task have ample reason to believe that feedback is not probabilistic, and thus that every trial has one correct and one incorrect answer. Unless otherwise indicated, we assume symmetrical updating.

Still missing from this algorithm is the *action policy*, the rule by which values *Q* are translated into actions. Our implementation of *Q*-learning makes use of the softmax decision rule (Sutton and Barto, 1998), originally due to Luce (1959). This gives the probability of taking an action *a*_*t*_, from the set of all actions available at that time *A*_*t*_, as follows:

(3) $$p\,\left(a_{t}|s_{t}\right)=\text{softmax}\,\left(Q,\theta \right)=\frac{\text{exp}\,\left(\theta \cdot Q\,\left(a_{t}|s_{t}\right)\right)}{\sum_{i\in A_{t}}\exp\left(Q\,\left(i|s_{t}\right)\right)},\theta \geq 0.0$$

This action policy is governed by one parameter θ, which determines how strongly the response alternatives contrast with one another. When θ is large, the policy responds almost exclusively to the choice with the highest expected value. As θ gets smaller, the odds of non-maximizing behaviors increase, until θ = 0.0 yields equiprobable chance responding.

### The Value Transfer Model: Value by Association

Following the demonstration of TI on monkeys by McGonigle and Chalmers (1977), there was heated debate over the correct definition and implications of “animal intelligence” (Macphail, 1987). TI in non-human animals was particularly provocative because it could not be easily explained by the reward associations that had dominated behavior analysis (and thus animal psychology generally) in the preceding decades. The “Value Transfer Model” (VTM) was proposed as a counterargument against a cognitive account (von Fersen et al., 1991), ostensibly providing an associative account of how TI might arise.

Under VTM, stimuli become associated with rewards according to a Rescorla–Wagner mechanism, and the strength of this association governs the relative rate at which stimuli are selected. However, they *also* accrue value from the associative strength of the stimuli they are paired with. In other words, value “transfers” between stimuli that appear together. Like most behavior-analysis studies of its time, this was demonstrated by first collecting data and then fitting a function to the resulting summary statistics. Under this analysis, VTM appears able to describe behavior in the classical experimental demonstrations of TI in animals (Wynne, 1995). However, VTM was *not* originally conceptualized as a process model that could be used in simulation.

We implemented VTM as a process model using the design described by Kumaran et al. (2016). This builds directly on the retrospective formalism described in Equation 2, and introduces an additional term τ to the value updating function:

(4) $$Q\,\left(a_{t}|s_{t}\right)\leftarrow Q\,\left(a_{t}|s_{t}\right)+\delta \,\left[c_{t}+\tau \,\left(\sum Q\,\left(\neg \,a_{t}|s_{t}\right)\right)-Q\,\left(a_{t}|s_{t}\right)\right]\alpha \in \left(0,1\right),\tau \in \left(0,1\right)$$

Here, the parameter τ corresponds to rate of “transfer” between stimuli and ∑*Q*(¬*a*_*t*_|*s*_*t*_) refers to the sum of all values of *Q* for the actions that were available to the actor but were *not* chosen. If τ = 0.0, the function is identical to Equation 2. As τ grows, the value of any given *Q* grows merely by association with the other presented stimuli, *independent* of the consequences of choosing *a*_*t*_. This implementation uses the softmax rule (Equation 3) as its action policy. VTM may be implemented symmetrically (updating every response option) or asymmetrically (updating only the chosen item). Unless otherwise noted, we used symmetrical updating.

Although some variant of VTM is typically presented as the go-to “behaviorist alternative” to cognitive theories of TI (e.g., André et al., 2012), VTM makes incorrect predictions under a wide range of experimental preparations (e.g., Weaver et al., 1997; Daniels et al., 2014; Vasconcelos and Monteiro, 2014). A particularly important failure was reported by Lazareva and Wasserman (2006, 2012), who introduced a block of trials between training and testing that consisted exclusively of massed presentation of a single pair of stimuli. This design did not undermine performance by subjects, despite VTM’s predictions that there should be a dramatic increase in error rates. We demonstrate this failure, among others, in the simulations below.

### REMERGE: Value by Configural Similarity

Although VTM is the most commonly cited associative model of TI, another approach was proposed at about the same time. Couvillon and Bitterman (1992) proposed that, rather than TI arising from cross-talk between reward associations, it could instead be explained by the similarity of the contexts in which behavior was occurring (i.e., the stimulus pairings). Under this “configural” theory of TI, subjects simultaneously learn about response alternatives *and* about stimulus pairs. The configural similarity of stimuli provides clues that could be used to exceed chance performance for novel pairs.

This theme of context configuration has been a long-standing proposal in computational neuroscience for how spatial or temporal processing might be implemented in the hippocampus (Wu and Levy, 1998; Rodriguez and Levy, 2004). In the context of the TI task, its most fully realized form is the REMERGE model, due to Kumaran and McClelland (2012).

**FIGURE 3:**
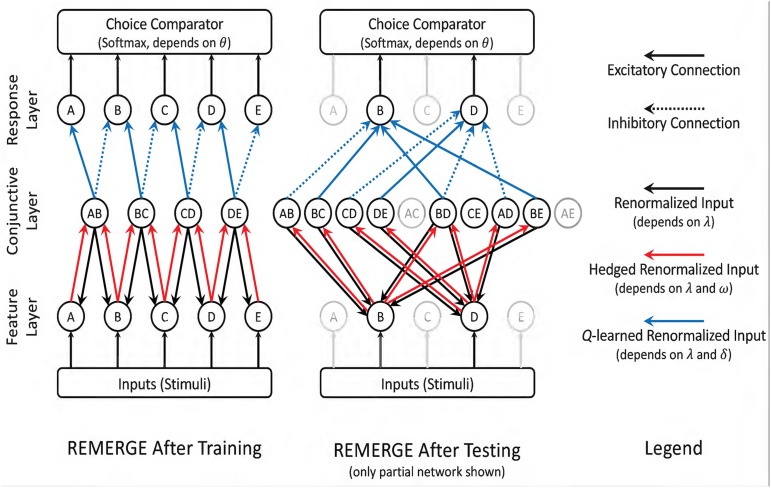
Schematic depiction of RL-REMERGE. Nodes in the Feature layer are initially stimulated by inputs (i.e., if a stimulus is present, its Feature node is excited). All solid-line connections are excitatory, whereas dotted-line connections are inhibitory. When stimuli in the Feature layer are excited, this flows through excitatory connections to the Conjunctive layer, with one node for each pairing the algorithm has previously observed. Excitation then flows back to the Feature layer, and so forth. Excitation entering the Conjunctive layer (red arrows) is “hedged” during renormalization, leading to dissipating excitation for nodes that are many steps removed from environmental input. Once the feedback between the Feature and Conjunctive layers has stabilized, excitatory and inhibitory inputs to the Response layer are summed, and a choice is made using the softmax rule. Because REMERGE is initially ignorant about the values between the Conjunctive and Response layers, those arrows (in blue) begin with values of 0.0 and their excitatory or inhibitory strength is subsequently discovered using *Q*-learning. In this scenario REMERGE learned a 5-item list. **(Left)** Following training, the Conjunctive layer only has four nodes, corresponding to the four adjacent pairs. This is sufficient to infer that B > D because information flow between the Feature and Conjunctive layers when B and D are externally stimulated yield a higher overall level of excitation for B than it does for D. **(Right)**. Partial network depicting *only* those nodes that are directly connected to options B and D. Since all ten pairs have now been observed, every stimulus in the Feature layer has links to four Conjunctive layer nodes. In practice, information flow still passes through all Feature and Conjunctive nodes, but exposure to all stimulus pairs makes that diagram too complex to depict in a figure. Reproduced with permission (DOI: 10.6084/m9.figshare.7992011.v1). Copyright 2019, Jensen, Terrace, and Ferrera.

REMERGE is a neural net model with three layers, as depicted in Figure 3. The “Feature layer” receives inputs from the environment, with each item in the list receiving its own node. The “Conjunctive layer” consists of one node for each stimulus pair. Each Feature node excites every Conjunctive node associated with a pair that its stimulus belongs to, *and* each Conjunctive node stimulates both Feature nodes associated with its pair of items. Meanwhile, activity in the Conjunctive layer stimulates nodes in the “Response layer,” with one node associated with each response alternative. Each Conjunctive node sends excitatory signals to the Response node associated with a correct response while also sending an inhibitory signal to the Response node associated with an incorrect response. The mutually excitatory feedback between the Feature and Conjunctive layers is renormalized until a steady state is reached, and at that point the relative activity of nodes in the Response layer provide the basis for the action policy to select one of the stimuli. The details for this implementation are given in the Supplementary Material.

As described by Kumaran and McClelland (2012), REMERGE has two free parameters governing the activity of the network; λ and ω govern the behavior of the feedback between network nodes. λ denotes the “temperature” of the network, which governs how much activity in the network is permitted to vary as it converges toward a steady state. ω corresponds to a constant “saturation” variable that determines how much hedging should occur in the activity of the conjunctive layer, effectively putting an upper limit on the resulting renormalized values. Kumaran and McClelland (2012) also give one free parameter that governs choice behavior, the θ parameter of a softmax action policy. This policy is identical to that given by Equation 3, except that instead of using *Q*, REMERGE applies softmax to the activity associated with all nodes in the Response layers that are available to the actor during the current trial. Our symbols λ, ω, and θ correspond to the parameters given by Kumaran and McClelland as $\frac{1}{\tau }$, *C*, and β respectively, but we have elected not to use their notation to minimize confusion with other symbols used in this manuscript.

Despite being a model of TI, REMERGE was *not* a model of learning in its original presentation, because the arrangement of the network presumed that training was complete and sought only to predict the actor’s behavior on the next trial. In order to make REMERGE a learning model, we made two changes. The first change was that each node in the Conjunctive layer was excluded from calculation until the first trial in which that stimulus pair was presented. Thus, given training only on adjacent pairs, REMERGE would only be able to make inferences using those pairs. This explains the difference between the two versions of the network depicted in Figure 3. Following training, the Conjunctive layer has only four nodes because it has only been exposed to four combinations of stimuli. Contrastingly, after testing, the Conjunctive layer includes a node for each of the ten possible pairs. This is because by that time the network has been exposed to all of those explicit cases.

Our second change was that rather than hard-coding the links between the Conjunctive and Response layers with the correct and incorrect responses, we require that REMERGE learn the values of those links by *Q*-learning (where incorrect responses were given a value of −1, rather than 0, to ensure incorrect links would become inhibitory), with a corresponding learning rate δ. Thus, our “reinforcement learning” version of REMERGE had four free parameters in total.

REMERGE straddles the line between model-free and model-based learning. Although it does not implement a model of the list *order*, it does implement a kind of logical syllogism. As Figure 3 depicts, the presentation of the pair BD allows these two stimuli to be indirectly associated by information flowing back and forth through the Feature and Conjunctive layers (following the path B ↔ BC ↔ C ↔ CD ↔ D). Importantly, however, this syllogism is subject to error propagating through the network. In particular, due to the hedging term ω, activity flowing along longer sequences of nodes (i.e., inferences involving response pairs with a larger symbolic distance) tends to wash out. The obvious prediction that follows from this is that REMERGE should display a *negative* symbolic distance effect at transfer, the opposite of the pattern observed in the empirical data (e.g., in Figure 2).

## Model-Based Solutions: Inference About Ordering

At the core of the debate over whether TI is better explained by model-free or by model-based learning is a disagreement about parsimony. Advocates of model-free TI (such as VTM) see the invocation of “mental representations” as a needless complication, whereas advocates of cognitive models argue that even though they are generally more computationally complex, they are nevertheless more plausible in light of their generality, as well as support from a host of experimental studies (Terrace, 2012).

Despite this, most cognitive models of serial learning have been conceptual in nature, consisting of qualitative descriptions of processes that give rise to group averages. Much of the empirical support cited for these theories relies on the failure of associative models to account for the available experimental results (Gazes et al., 2012). Assessing the complexity of these cognitive theories is difficult because most cognitive models of serial learning have not been characterized in computational terms. This paucity of model-based learning algorithms that are based on cognitive theories may be in part due to the difficulty of designing a plausible model-based algorithm for *learning* TI that does not presuppose the list order. As such, in order for a model-based algorithm to provide a satisfactory description of serial learning, it must be able to begin with novel stimuli, deduce the correct order given some pattern of stimulus pairings, and display the characteristic pattern of errors seen in real subjects while doing so.

First, it is important to characterize the range of possibilities for a model-based algorithm. Since TI is presumed to be an inference about the order of the items in a list, an algorithm that bases its response on *any* model of ordering, rather than on expected value, may be considered a cognitive model. The simplest such model is simply an ordered list that is able to adjust its ordering on the basis of feedback.

### RL-Elo: Semiparametric Rank Estimation

The Elo rating system was first developed for use in chess (Elo, 1978) and has since been used in a variety of other competitive settings. Each competitor has a rating (e.g., Garry Kasparov retired from chess with a rating of 2812), which can be compared to the rating of others in order to predict who is more likely to win in a match-up. When a sanctioned match between players occurs, the amount by which each player’s score changes is a function of how surprising the result is, relative to the rating’s prediction. Although designed for human competition, the dynamic character of the Elo rating system (able to change over time as new information comes in) has made it a popular model for other scientific problems of ordinal rank, such as the analysis of dominance hierarchies in animal groups (Neumann et al., 2011).

To adapt this approach to the problem of TI, Kumaran et al. (2016) implemented a reinforcement learning version of this system, which they called “RL-Elo.” Under this system, each stimulus was assigned a score of 0.0 in a vector *V* at the start of training, and choices were made according to the softmax rule (Equation 3) applied to the elements of *V* that were associated with the stimuli visible during each trial (with a corresponding free parameter θ). When ratings are evaluated on a pairwise basis (e.g., *a*_*t*_ and ¬*a*_*t*_), this reduces to the logistic function, defined in terms of the difference between the two values of *V*:

(5) $$p\,\left(a_{t}\right)=\frac{1}{1+\exp\left(-\theta \,\left(V\,\left(a_{t}\right)-V\,\left(\neg \,a_{t}\right)\right)\right)}$$

This action policy is effectively the softmax rule (Equation 3), albeit limited to two alternatives. However, rather than updating the stimuli as a function of their expected value, RL-Elo updates them with respect to their expected probability of a correct response. Thus, if *a*_*t*_ is the correct response, then given the probability that a correct response *p*(*a*_*t*_),

(6) $$V\,\left(a_{t}\right)\leftarrow V\,\left(a_{t}\right)+\delta \,\left(1-p\,\left(a_{t}\right)\right)$$

$$V\,\left(\neg \,a_{t}\right)\leftarrow V\,\left(\neg \,a_{t}\right)+\delta \,\left(p\,\left(a_{t}\right)-1\right)$$

δ∈(0,1)

**FIGURE 4:**
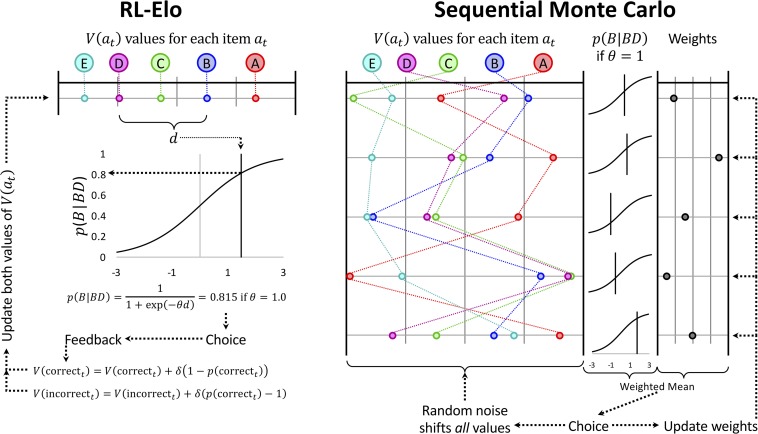
Schematic depiction of the updating rules used for the RL-Elo and Sequential Monte Carlo (SMC) algorithms. **(Left)** RL-Elo represents each list item *a*_*t*_ as a stimulus as having a value *V*(*a*_*t*_) along some unitless continuum. The probability of choosing the item is obtained by computing the difference *d* between that item and its alternative (*d* can be negative if the item has the lower of the two values) and passing *d* through the logistic function. The parameter θ adjusts the contrast. If the choice made was correct, its value is adjusted as a function of how far that value was from its expectation, adjusted by a learning rate δ. **(Right)** The updating process during a single trial for SMC is demonstrated using a sample of five hypothetical orderings (out of the 10,000 that our implementation used). As with RL-Elo, each difference score yields a probability of selection using the logistic function. Additionally, each hypothetical ordering has an associated weight based on how well it has predicted previous trials. The choice is made on the basis of a weighted average of the probabilities, and the feedback is used to update the weights (strengthening or weakening each ordering’s influence as a function of how well it predicted the outcome of the trial). The orderings themselves are then modified using Gaussian noise. Not shown is the algorithm periodically throwing out the existing 10,000 orderings in favor of a new set of alternatives. Reproduced with permission (DOI: 10.6084/m9.figshare.7992014.v1). Copyright 2019, Jensen, Terrace, and Ferrera.

Here, the learning rate δ makes another appearance, but this time it is being used to update the *score* associated with a stimulus, without consideration for its expected value. Consequently, the engine that powers RL-Elo is not expected value, but rather expected difference in rating. Under this implementation, RL-Elo updates stimuli symmetrically. Since this difference is effectively tracked as a difference on the logit scale, the vector *V* can be seen as a semiparametric model for estimating the ranks of the stimuli. From this perspective, *V*(*a*_*t*_) makes no distinction between a representation of the rank of a stimulus and the preference for selecting that stimulus during trial *t*. The result is a gradient-following algorithm (Williams, 1992) that adjusts the scores in *V* until such time as difference in the scores between any two items yields close to perfect discrimination of their rank. The logic of this updating scheme is depicted in Figure 4 (left).

### Sequential Monte Carlo: Iterative Crowdsourcing

Most statistical analyses of ordinal rank adopt a semiparametric strategy, as RL-Elo does. However, estimating some scalar quantity as a proxy for the ordinal rank has limitations. One is that, like most model-free strategies, adjusting the values associated with stimuli only after they were presented can be unreliable when some item pairs are presented more often than others (i.e., unequal base rates of presentation). That limitation is overcome by “Sequential Monte Carlo” (SMC) algorithms (Kumaran et al., 2016). Sometimes called “particle filter” algorithms, these provide an approximation to Bayesian reasoning about ordinal rank similar to the approach used by a Kalman filter (Doucet et al., 2000). The details of its implementation are given in the Supplementary Material.

Rather than consider a single vector of scalar values, as RL-Elo does, SMC simultaneously considers a very large number of them. Our implementation uses 10,000 such “hypothetical orderings,” generated randomly from a normal distribution (with variance $\sigma_{\emptyset }^{2}$) at the outset of training. Rather than adjust those orderings adaptively based on feedback, the values of all items in all hypothetical orderings are adjusted at random by adding normally distributed random noise (with variance σ^2^) to each value. In general, σ_∅_ is large relative to σ, in order to ensure that the various hypothetical orderings differ considerably from one another. SMC does not use the feedback to update the orderings directly, but instead uses it to update the *weight* associated with each hypothetical ordering. All orderings begin with equal weights, but over time, those whose predictions are consistent with feedback are given more weight, while those that are not consistent are given less weight. Orderings that happen by chance to be mostly correct come to dominate the overall representation of order, while those that make consistently poor predictions are reduced to having almost no influence. The choice that the actor then makes is based on the weighted average of the estimated stimulus positions, based on a softmax decision rule (Equation 3) with θ as a free parameter. This implementation thus has three free parameters: σ_∅_, σ, and θ.

SMC can be seen as 10,000 RL-Elo estimates, except that all stimuli are being updated at random all the time. Those orderings that are consistently right are given greater weight than those that are consistently wrong, and it is the *weight* that is updated on the basis of feedback, using the delta rule given in Equation 6. Rather than trying to update a single ordering optimally (as RL-Elo does), SMC updates a crowd of possible orderings randomly, and then updates the “fitness” or “reputation” of those random orderings on the basis of feedback to separate out the good vs. the bad models. Figure 4 (right) gives a schematic example of this updating process (albeit using only five hypothetical orderings, rather than 10,000).

Even with 10,000 simultaneous models, however, there is a risk that *none* of the hypothetical orderings are ordered correctly. We know that monkeys can learn 9-item lists (Jensen et al., 2013) and 15-item lists (Treichler et al., 2003), but for lists of those lengths there are 9! and 15! (i.e., 362,880 and 1.3 trillion) possible orderings, respectively. As such, SMC includes a criterion to resample its list orderings periodically, generating a new set of 10,000 hypotheses using its existing 10,000 hypotheses and their respective weights as a seed. Put another way, the new set of 10,000 hypotheses are drawn, with replacement, from the previous set of 10,000, using the learned weights. Thus, orderings that have close to zero weight have little chance of being resampled, whereas those with high weight are likely to appear multiple times in the new generation of hypotheses (after which they diverge due to the added noise).

SMC algorithms give reasonable approximations to behavior in a variety of contexts (Yi et al., 2009). Insofar as they constitute a Monte Carlo estimation process, filters with a sufficiently large number of particles should converge on optimal estimates given sufficient evidence. SMC’s periodic resampling allows the algorithm to weed out bad hypotheses and gradually converge on a high-precision estimate of the list ordering, giving it an advantage over a conventional Kalman filter. However, this advantage relies on the assumption that the target ordering is static. In the event that the ordering changes, this resampling can result in the algorithm painting itself into a corner, from which it can only gradually escape. Consequently, a reasonable prediction is that SMC should be robust to many experimental manipulations, but should respond poorly to experimental designs that change the ordering of the list.

### Betasort: Modeling Position, Uncertainty, and Transitivity

Although score-differential models of rank like RL-Elo and SMC have the potential to be effective in sorting well-ordered lists, they have a number of limitations. The most substantial of these is that they assume that the uncertainty of each score is uniform. If, for example, a subject is trained extensively on a five-item list ACDEF, and is only later introduced to the stimulus B, those models have no way of representing that the position of B is much more uncertain than the positions of the other stimuli. To fix this, the position of each stimulus would need to be represented by a minimum of two parameters. Attempts to extend the Elo rating system directly, such as Glickman’s “Glicko” and “Glicko-2” algorithms (described by Samothrakis et al., 2016) provide a framework for introducing additional parameters, but their additional model complexity can create scenarios in which ratings become trapped in local minima, as well as relying on hyperparameters that must be set *ad hoc* to govern how dispersion evolves over time. It would be preferable to keep action selection and value updating be as simple and as computationally efficient as possible.

Jensen et al. (2015) proposed the “Betasort” algorithm as a computational model of serial learning. Under this framework, the position of each item in the list is represented by a beta distribution whose shape is determined by two values, *U* and *L* (corresponding to the “upper” and “lower” ends of a unit scale). An item’s probability density *D*(*x*) between 0.0 and 1.0 is given by the following formula:

(7) $$D\,\left(x\right)=\text{BetaPDF}\,\left(x|U,L\right)=\frac{x^{U-0.5}\cdot {\left(1-x\right)}^{L-0.5}\,\Gamma \,\left(U+L+1\right)}{\Gamma \,\left(U+0.5\right)\cdot \Gamma \,\left(L+0.5\right)}$$

Note that BetaPDF(*x*|*U*,*L*) assumes the inclusion of a Jeffrey’s prior, which introduces a slightly conservative factor to the estimate that facilitates numerical estimation. This is accomplished by adding a value of 1/2 to each term, on the principle that the beta distribution is its own conjugate prior, such that BetaPDF(*x*|*U* + 0.5,*L* + 0.5)∝BetaPDF(*x*|*U*,*L*)BetaPDF(*x*|0.5,0.5). Although it would be most appropriate to say that the entire distribution *D*(*x*) represents the estimate of an item’s position, a summary is given by the mean, $\frac{U+0.5}{U+L+1}$.

The action policy under Betasort is to draw a random value from each distribution that corresponds to an available action, and to take the action with the largest value. The net effect of a random draw procedure is a baseline probability of choosing an action *a*_*t*_ given by the following formula (Raineri et al., 2014):

(8) $$p_{\text{beta}}\,\left(a_{t}\right)=\int_{0}^{1}\text{BetaPDF}\,\left(x,U_{a_{t}},L_{a_{t}}\right)\cdot \left[\int_{0}^{x}B\,e\,t\,a\,P\,D\,F\,\left(y,U_{\neg \,a_{t}},L_{\neg \,a_{t}}\right)\,𝑑y\right]\,𝑑x$$

Although determining the value of this double integral is computationally prohibitive, doing so is not necessary to simulate behavior using this procedure. This is because making random draws from two beta distributions is much less computationally intensive than solving the integral above.

**FIGURE 5:**
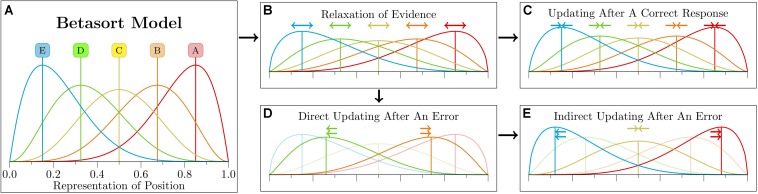
Schematic depiction of the Betasort algorithm’s representation of position. Quantitative details about each step are described by Jensen et al. (2015). **(A)** Each stimulus has an associated beta distribution, whose parameters encode both the stimulus position and its uncertainty. In order to choose between available options, the algorithm draws a value for each possibility, then chooses the item whose random draw yielded the highest value. Additionally, there is a probability ρ of the subject ignoring these values and making a response at random. **(B)** Following a choice, the representation is updated. The first stage of updating is “relaxation,” which increases the uncertainty of the estimated position without changing the means of any of the distributions. This applies to all items, regardless of whether the stimuli were present during that trial or not. **(C)** If the subject makes a correct choice, then the distributions for all items are consolidated (again, whether or not any given stimulus was presented). What subject make consistently correct responses, the relaxation and consolidation steps effectively balance one another out. **(D)** If, however, the subject makes an incorrect choice, then updating proceeds in two stages. During direct updating, the means of the stimuli presented during that trial are moved apart to reduce the odds of another erroneous response. **(E)** The preceding step is them immediately followed by incorrect updating of all stimuli that were *not* visible during the trial. Stimuli that were between the two items are consolidated, items whose current mean lies below that of the pair are shifted further downward, and pairs whose mean lies above the pair are shifted upward. Reproduced with permission (DOI: 10.6084/m9.figshare.7992017.v1). Copyright 2019, Jensen, Terrace, and Ferrera.

Unlike RL-Elo and SMC, Betasort applies its value updating function to all stimuli (including those that are not presently visible) and treats positive feedback differently from negative feedback. Figure 5 gives a visual sense of these updating steps. Following every trial, the existing values of *U* and *L* are relaxed by a “recall” parameter φ, whose value is less than 1.0. This effectively makes *U* and *L* leaky accumulators (Grice, 1972), although in Betasort’s case, the strength of the forgetting imposed by φ is further adjusted as a function of how frequently the algorithm makes correct responses. See Jensen et al. (2015) or the Supplementary Materials for details.

If a choice yielded positive feedback, every value of *U* is increased by $\frac{U}{U+L}$ and every value of *L* is increased by $\frac{L}{U+L}$. This has the effect of increasing the model’s confidence of all position estimates, because it reduces the variability of all position distributions without shifting any of the distribution means. If, on the other hand, the feedback was negative, several updates happen in succession. First, the chosen item has its *L* increased by 1 while the item that was not chosen has its *U* increased by 1. Next, all of the stimuli that were *not* chosen have their values updated as well. Items above the visible stimulus pair have their values of *U* increased by 1, items below the pair have their values of *L* increased by 1, and those falling in between have their positions consolidated as though the feedback had been positive. This “implicit updating” stage ensures that, in general, the full list will preserve its ordering. As demonstrated below, implicit updating is needed to explain why experimental designs like the massed trials studies by Lazareva and Wasserman (2006, 2012) do not yield preference reversals.

One of the commonly reported features of TI studies, particularly those involving non-human animals, is a background error rate that persists even after extensive training. Whether mistakes are made due to memory errors or to shifting attention, they are not captured by models that converge on perfect discrimination. Consequently, Betasort introduces a “random response” parameter ρ, which is the probability that the subject, on a given trial, ignores its current knowledge and instead makes a response at random. This could also be considered a “lapse rate,” governing the frequency with which the subject lapses into making an uninformed response. When taking this additional factor into consideration, the probability of choosing *a*_*t*_ given by Equation 7 is updated in the following way:

(9) $$p_{\text{beta}}\,\left(a_{t},\rho \right)=\frac{\rho }{2}+\left(1-\rho \right)\cdot p_{\text{beta}}\,\left(a_{t}\right)$$

The ρ parameter effectively puts a ceiling on performance and scales the remaining probabilities toward chance.

## Simulation of Serial Order Tasks

The algorithms described in the previous section are process models, and their behavior evolves over the course of training. One of the consistent themes of using process models to describe natural processes is that their results are often contrary to our intuitions. As such, the evaluation of these algorithms is best performed by simulating the consequences of various experimental designs and observing how they perform, rather than merely thinking about them intuitively. We subjected the algorithms to various experimental manipulations to demonstrate their relative efficacy and performance idiosyncrasies. In all cases, the figures below represent mean accuracies over 1000 simulations, each of which had its own randomized trial order of stimulus presentations.

### Parameter Selection

The algorithms each had 2–4 free parameters, which afforded varying degrees of flexibility in describing performance. Our objective was not to discover each algorithm’s optimal performance, but instead to use parameters that best described a real-data target. Put another way, rather than describe each algorithm’s normative performance, we tested how well each approximates an empirical estimate of TI performance in an actual organism. In particular, we were interested in those parameters that recapitulate “performance at transfer” in a classic TI design. With this in mind, we selected one monkey (named “Hubble”) whose TI data was published by Jensen et al. (2015). Likelihoods were calculated for the six algorithms with respect to the sequence of trials and the subject’s choices during the first 21 trials of all-pairs testing, immediately after the end of training. This ensured that, however, performance appeared early in training or late in testing, it resembled the critical transfer test as closely as possible.

In order to perform statistical inference using process models, the response likelihoods must also be calculated on a trial-by-trial basis, unfolding as each models’ representation is updated. Due to random processes that vary from one simulation to the next (particularly for SMC), calculations of the likelihoods were noisy. Additionally, the 3- and 4-parameter algorithms were not well suited to grid approximation. Consequently, maximum-likelihood parameters were obtained by quadratic approximation. As a shorthand, we will subsequently describe these as each algorithm’s “Hubble parameters.”

According to this procedure, *Q*-learning’s Hubble parameters were δ = 0.138, θ = 2.280, and γ = 0.373. As a shorthand, error prediction learning rates like δ can be thought of in terms of their inverse. That is, if δ = 0.138, then the actor will base its estimates on about the previous $\frac{1}{\delta }=7.2$ updates of that value. This is a relatively rapid learning rate, focused only on recent events. The decision rule θ = 2.280 corresponds to an exaggerated preference for whichever alternative has a large value of *Q* (i.e., “winner-takes-all”). For example, if *Q*_*A*_ = 1 and *Q*_*B*_ = 0.5, then chance of choosing A over B would only be 0.622 if θ = 1, whereas the probability for A would be 0.758 if θ = 2.280. A prospective discounting rate of γ = 0.373 would normally suggest an actor that values future states at about ^1^/_3_ of the value of current choices. However, since current choices in the TI task exert no control over future states, this term effectively introduces a small amount of noise into performance, shifting all behavior slightly closer to chance.

The Hubble parameters for VTM were δ = 0.137, θ = 2.260, and τ = 0.406. the learning rate and decision rule terms were similar to those obtained for *Q*-learning, namely relatively fast learning and an exaggerated preference for the stimulus with the maximum *Q* estimate. The transfer rate τ was relatively large. For each unit of feedback that was learned about the value of a stimulus, 0.406 units were also learned from the alternative with which it was paired.

The Hubble parameters for RL-REMERGE were λ = 2.690, ω = 15, θ = 4.971, and δ = 0.500. Because λ constituted the “inverse temperature,” higher values correspond to less volatility while the network approached equilibrium. The very large value of ω ensured that information flowing through the network washed out after only a few steps through the conjunctive layer. This is certainly bad for the actor’s performance, and was likely the most efficient way for REMERGE to approximate the persistent error rates observed in subjects after extensive training. The decision rule θ was quite large, indicating a strong preference for the Response node with maximal activity. The learning rate δ was very high relative to the other model-free algorithms. Although this would ordinarily make behavior highly volatile, the fact that the Conjunctive layer had a node for every observed stimulus pair meant that the algorithm was at an advantage because it could memorize pairs on a case by case basis. A high value of δ enabled the network to pick out and keep those contextually correct responses, even given only a few exposures.

The Hubble parameters for RL-Elo were δ = 0.079 and θ = 1.529. Although these values may seem smaller than those found for *Q*-learning and VTM, it is important to remember that the model-free algorithms are comparing rewards (1 in most cases) to non-rewards (0), whereas Elo is comparing wins (1) with losses (−1). Since the units for RL-Elo effectively cover twice the numerical range of the model-free algorithms, its units need only be half as big to have a comparable effect. Viewed from this perspective, RL-Elo’s parameters are quite similar to those of the model-free algorithms.

The Hubble parameters for SMC were σ_∅_ = 0.5, σ = 0.032, and θ = 1.410. The σ_∅_ parameter was used to initialize the 10,000 candidate orderings, and its relatively small value ensured that all of the candidates were relatively confusable with one another. Meanwhile, the even lower value of σ ensured that the amount by which these representations changed from one trial to the next was small. The net effect was that SMC’s prodigious ability to solve ordinal sorting problems was dampened, allowing it to better approximate Hubble’s gradual learning and tendency to make errors. The decision rule θ was very similar to that of RL-Elo, which is sensible because the two made similar use of a logistic discrimination function.

The Hubble parameters for Betasort were ρ = 0.188 and φ = 0.495. The randomness parameter ρ means that, for about every two trials out of eleven, Hubble was expected to give a random response regardless of the stimuli, effectively putting a ceiling on his performance. Meanwhile, the recall parameter φ had a very low value, suggesting that the representation lost roughly half of its evidence for each trial. This had the effect of keeping all of the stimulus positions uncertain, which increased the error rate but also increased how rapidly positions could be adjusted on the basis of new information.

### Classical Transitive Inference

The classic procedure for demonstrating TI is to set up an ordered list (e.g., ABCDEFG) and train subjects by presenting only the adjacent pairs of items (e.g., AB, BC, CD, DE, EF, and FG), giving positive or negative feedback for single responses. After training, the actor is tested with all possible pairs of items (21 in this case). Both training and testing consist of “blocks,” during which each stimulus appears a fixed number of times. Since it is standard to counterbalance the spatial arrangements on a screen (AB vs. BA), we presented the actor with each pair twice during a block. Thus, training blocks consisted of 12 trials (each pair appearing twice), with the order of trials in any given block randomly permuted. This guaranteed that the actor’s choice *a*_*t*_ had no influence on their state *s*_*t* + 1_. Training consisted of 11 blocks, and thus of 132 trials.

During testing, actors were exposed to all 21 possible item pairs (AB, AC, AD, etc.). As in training, each pair appeared twice per block, so testing blocks consisted of 42 trials, randomly permuted. Testing consisted of five blocks, and thus of 210 trials. The testing set includes six “critical” pairs (BD, BE, BF, CE, CF, and DF) which (1) were not presented during training and (2) do not include the terminal items A or G. In this and all subsequent simulations, rewards were delivered for correct responses during the testing phase, consistent with the data collected from Hubble.

**FIGURE 6:**
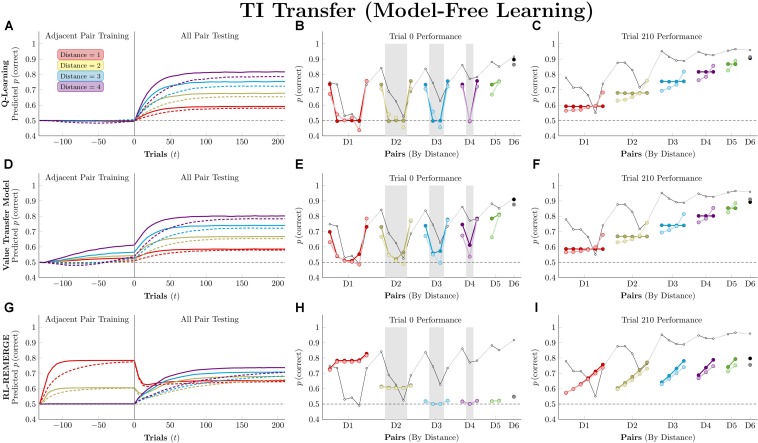
Performance of model-free algorithms on a 7-item TI transfer task (adjacent pair training, all pair testing). **(A)** Mean performance of *Q*-learning on non-terminal pairs throughout training and testing, sorted by symbolic distance. Solid lines denote learning with symmetric updating of both alternatives, whereas dashed lines denote asymmetric updating of values associated with the chosen stimulus. **(B)** Mean performance of *Q*-learning for each of the 21 pairs at transfer, sorted by symbolic distance. Open points are empirical estimates of performance by Hubble, a monkey reported by Jensen et al. (2015). Dark points correspond to symmetric updating, while light points denote asymmetric updating. Critical pairs are shaded in gray. **(C)** Mean performance of *Q*-learning for each of the 21 pairs at the end of training. Open points are empirical estimates of performance by Hubble. Dark points correspond to symmetric updating, while light points denote asymmetric updating. **(D–F)** As above, but reporting simulated performance of the Value Transfer Model (VTM). **(G–I)** As above, but reporting the simulated performance of RL-REMERGE. Reproduced with permission (DOI: 10.6084/m9.figshare.7992020.v1). Copyright 2019, Jensen, Terrace, and Ferrera.

Figure 6 (left column) depicts the mean predicted response accuracy by model-free algorithms for all pairs composed of non-terminal items (e.g., BC and BD, but not AB or EG), grouped by symbolic distance. Trial “zero” in this case consists of the state of knowledge after the last trial of training but before the first trial of testing, and thus represents estimated accuracy at transfer. Dashed lines correspond to asymmetric updating (in the tradition of behavior analysis), which generally display slower learning rates because asymmetric updating results in less evidence extracted from each trial. Figure 6 (middle column) displays the response accuracy for each of the 21 pairs at trial zero, with the critical pairs shaded in gray. Hubble’s estimated response accuracy for the 21 pairs is also plotted allowing comparison between the algorithms and the subject they are trying to approximate. Dark points correspond to symmetric updating, and light points correspond to asymmetric updating. Overall, this simulation confirms previous reports (Jensen et al., 2015, 2018) that *Q*-learning shows no TI at the start of testing and no learning on non-terminal pairs throughout training. VTM performance yielded a small transfer effect (with the critical non-adjacent pairs above chance), a positive symbolic distance effect, and a substantial terminal item effect when run using its Hubble parameters. Although the distance effect is real, it is much smaller than that displayed by Hubble. On this basis, it is fair to say that VTM can “solve” this TI task (insofar as accuracy on critical pairs exceeds chance), particularly when using symmetric updating, but still underperforms on pairs with large symbolic distances.

Superficially, RL-REMERGE also “solved” the TI task, insofar as some of its critical pairs were above chance at test. However, it displayed a negative symbolic distance effect and no clear terminal item effect. For pairs with a symbolic distance greater than 2, performance was close to chance. This is due to the high value of ω used in the simulations. Herein lies the essential tension in using REMERGE as an explanatory model: If ω is small, performance of proximate pairs (which are more numerous) will be much too high, whereas if ω is large, distant pairs of items will be close to chance. Since REMERGE is expected to display a negative distance effect at test in all cases, it cannot come to resemble empirical results in their particulars.

Figure 6 (right column) displays response accuracy at the end of training (on trial 210). Here, *Q*-learning and VTM appear nearly indistinguishable, both showing positive distance effects. RL-REMERGE provides an interesting contrast. Despite receiving symmetric feedback for item pairs, the network displays an effect of rank.

**FIGURE 7:**
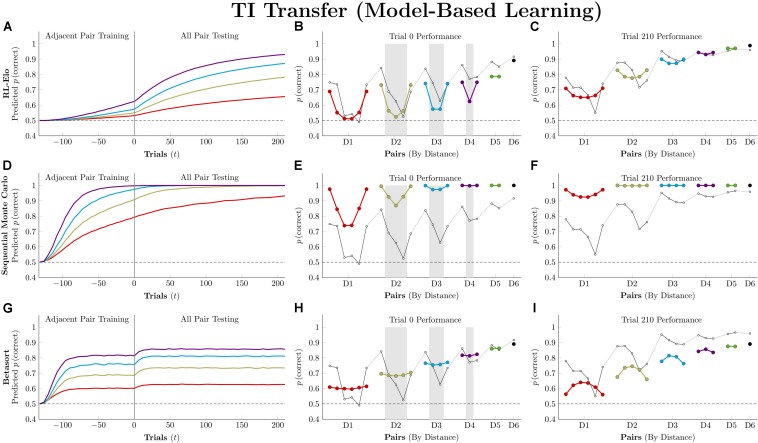
Performance of model-based algorithms on a 7-item TI transfer task (adjacent pair training, all pair testing). **(A)** Mean performance of RL-Elo on non-terminal pairs throughout training and testing, sorted by symbolic distance. **(B)** Mean performance of RL-Elo for each of the 21 pairs at transfer, sorted by symbolic distance. Open points are empirical estimates of performance by Hubble, a monkey reported by Jensen et al. (2015). Critical pairs are shaded in gray. **(C)** Mean performance of RL-Elo for each of the 21 pairs at the end of training. Open points are empirical estimates of performance by Hubble. **(D–F)** As above, but reporting simulated performance of the Sequential Monte Carlo (SMC) algorithm. **(G–I)** As above, but reporting the simulated performance of Betasort. Reproduced with permission (DOI: 10.6084/m9.figshare.7992023.v1). Copyright 2019, Jensen, Terrace, and Ferrera.

Figure 7 plots the simulation results for the three model-based algorithms, all of which show evidence of TI at the start of testing. Of these, RL-Elo appears to provide the closest approximation to the qualitative features of the data (showing both a positive distance effect and a terminal item effect), as well as a close approximation to the performance at trial 210. However, its performance at transfer is too low for many of the pairs. Meanwhile, SMC also displays both distance and terminal item effects, but its performance is too high in all cases. Finally, Betasort provides a good approximation to the *overall* response accuracy at test (including distance effects), but does not give a clear terminal item effect. Furthermore, its terminal item effect is negative at trial 210, a result not seen in empirical TI results.

### Distorting the Expected Reward Gradient

Model-free learning relies on comparisons of expected value as a proxy for preference. If different stimuli are associated with the delivery of different amounts of reward (i.e., to have different “reward magnitudes”), model-free judgments of preference should be badly disrupted if the reward information is not concordant with the optimal preference ordering. The worst-case scenario is a “reversed reward gradient,” in which an item’s rank also corresponds to its quantity of reward. So, stimulus A is always correct because its rank is 1, but is only worth 1 unit of reward. By contrast, F has a rank of 6, and as such is worth 6 units of reward in the pair FG, but is incorrect (and thus worth nothing) in the pairs AF, BF, CF, DF, and EF. Jensen et al. (2018) report that monkeys are able to solve TI tasks in spite of a reversed reward gradient, while *Q*-learning was unable to solve the problem in all cases regardless of the parameters that were used.

**FIGURE 8:**
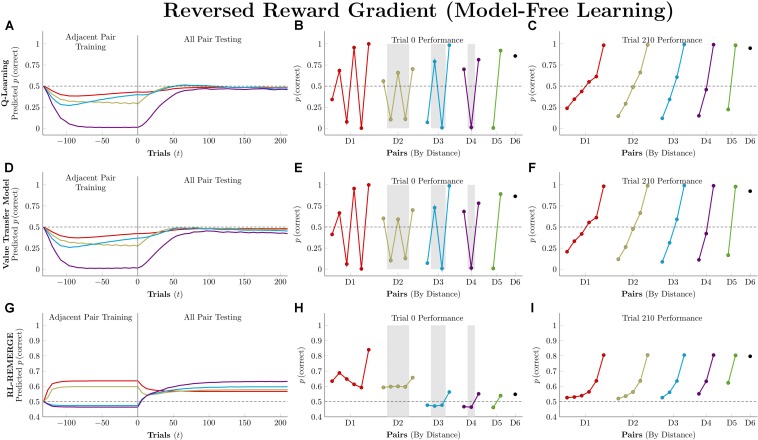
Performance of model-free algorithms on a 7-item TI transfer task (adjacent pair training, all pair testing) in which the amount of reward delivered corresponded to the rank of the correct response (i.e., 1 unit for A, 2 units for B, and so forth). **(A)** Mean performance of *Q*-learning on non-terminal pairs throughout training and testing, sorted by symbolic distance. **(B)** Mean performance of *Q*-learning for each of the 21 pairs at transfer, sorted by symbolic distance. Critical pairs are shaded in gray. **(C)** Mean performance of *Q*-learning for each of the 21 pairs at the end of training. **(D–F)** As above, but reporting simulated performance of the Value Transfer Model (VTM). **(G–I)** As above, but reporting the simulated performance of RL-REMERGE. Reproduced with permission (DOI: 10.6084/m9.figshare.7992026.v1). Copyright 2019, Jensen, Terrace, and Ferrera.

Figure 8 depicts performance of the model-free algorithms in an experimental procedure that is identical to that described in the preceding sections (7-item list, 11 blocks of adjacent-pair training, 5 blocks of all-pair testing, each pair presented twice per block). Because it would not be reasonable for actors to know the amount of reward associated with each stimulus in advance, these simulations were performed using asymmetric updating of only the chosen item.

For both *Q*-learning and VTM, response accuracy on the critical pairs is badly disrupted, but the resulting disruption takes on a surprising form: A sawtooth of alternating high-value and low-value estimates for pairs at each symbolic distance. This pattern is a consequence of the asymmetric updating during training. The value of F is driven very high by its hefty 6-unit rewards, and this causes it to be strongly preferred over E. As a consequence, D in the pair DE is also preferred. If all rewards were equal, then feedback during EF trials would cancel this effect out, but F’s larger rewards tend to overshadow the smaller values earned for selecting E (which happens less and less often as F grows in value). The overall effect is near-exclusive selection of F and an alternating pattern of preference propagating down the list, gradually becoming washed out by the time AB is being considered. Once testing begins and all pairs start being presented, this effect rapidly washes out and subjects instead come to favor later list items in general. Because G never accrues any worth, pairs that include it remain above chance, but the remaining stimuli are consistently distorted by their reward values, causing many pairs to be selected at below-chance rates throughout testing.

RL-REMERGE displays a strikingly different pattern that is much more robust against this manipulation. Although some pairs are pushed slightly below chance during training, the algorithm is broadly resistant to the intervention, maintaining response accuracies above chance for all pairs by the end of testing. This is likely because the configural character of the REMERGE network prioritizes contextual information (“When BC, choose B”), and only reflects global information through its network propagation (which rapidly washes out when ω is large).

Model-based algorithms were not run in this condition because they do not adjust their behavior on the basis of reward magnitude, only outcome (correct or incorrect). As a consequence, their predicted behavior would be exactly the same as that described in Figure 7.

Model-free learning algorithms fail this task dramatically. Not only does overall learning remain flat during training, performance on 11 of the 21 pairs remains below chance even after 210 trials of testing. Because reward does not provide a proxy for stimulus rank in this procedure, an organism that is able to solve this problem cannot be operating on the basis of model-free learning alone (Jensen et al., 2018).

### Massed Presentation of a Single Stimulus Pair

Studies of TI almost universally present the various stimulus pairings with equal frequencies, and this procedural assumption underlies many results claiming support for particular models (e.g., Wynne, 1995). However, such models can diverge from the behavior of organisms when a particular pair is presented *en masse* (Lazareva and Wasserman, 2006, 2012; Jensen et al., 2017). These “massed presentation” designs constitute a vital stress-test for models, because feedback in natural environments is almost never counterbalanced.

Actors in this simulation were first trained on 132 trials of adjacent pairs, as described previously. Then, they were trained for an additional 132 trials on only a single pair: FG. After both phases of training, actors completed 210 trials (5 blocks) of all-pairs testing.

**FIGURE 9:**
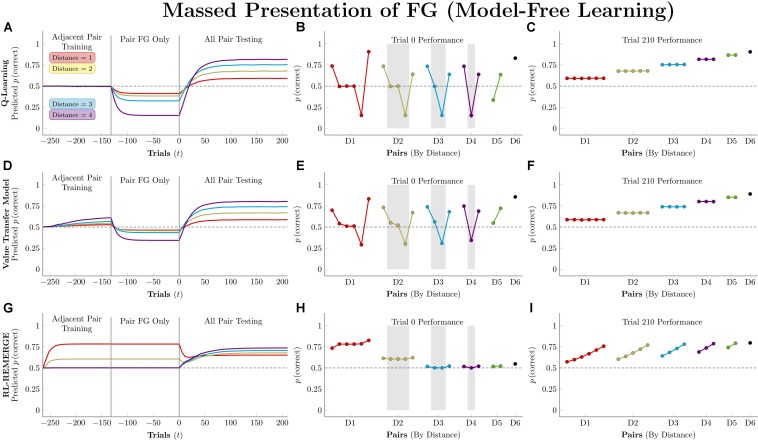
Performance of model-free algorithms on a 7-item TI transfer task (adjacent pair training, all pair testing) with an intervening period during which only the pair FG was presented. **(A)** Mean performance of *Q*-learning on non-terminal pairs throughout training and testing, sorted by symbolic distance. **(B)** Mean performance of *Q*-learning for each of the 21 pairs at transfer, sorted by symbolic distance. Critical pairs are shaded in gray. **(C)** Mean performance of *Q*-learning for each of the 21 pairs at the end of training. **(D–F)** As above, but reporting simulated performance of the Value Transfer Model (VTM). **(G–I)** As above, but reporting the simulated performance of RL-REMERGE. Reproduced with permission (DOI: 10.6084/m9.figshare.7992029.v1). Copyright 2019, Jensen, Terrace, and Ferrera.

Figure 9 depicts the consequences of massed presentation of FG for model-free algorithms. Both *Q*-learning and VTM have their performance disrupted by this manipulation, manifesting in reduced accuracy on other pairs that include F, but otherwise leaving their representations intact. VTM’s disruption was smaller than that of *Q*-learning, because F’s association with the valueless stimulus G dampens its expected value, even though F was correct for 132 consecutive trials. RL-REMERGE, however, was completely unaffected by massed trials, since the extended training on FG had no impact on the excitatory/inhibitory connections of other pairs in the Conjunctive layer.

**FIGURE 10:**
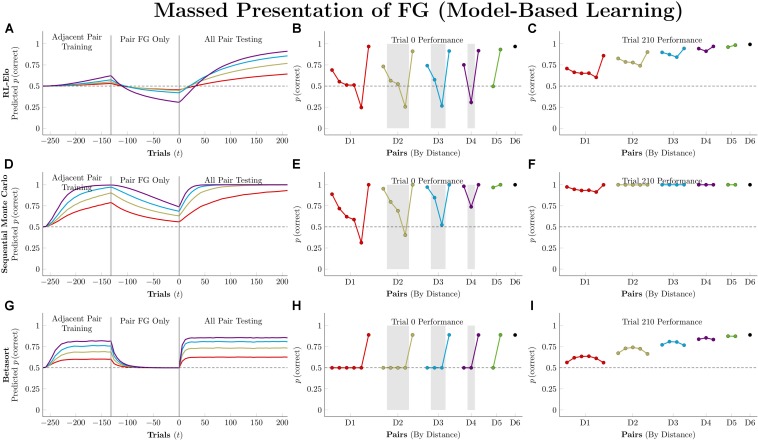
Performance of model-based algorithms on a 7-item TI transfer task (adjacent pair training, all pair testing) with an intervening period during which only the pair FG was presented. **(A)** Mean performance of RL-Elo on non-terminal pairs throughout training and testing, sorted by symbolic distance. **(B)** Mean performance of RL-Elo for each of the 21 pairs at transfer, sorted by symbolic distance. Critical pairs are shaded in gray. **(C)** Mean performance of RL-Elo for each of the 21 pairs at the end of training. **(D–F)** As above, but reporting simulated performance of the Sequential Monte Carlo (SMC) algorithm. **(G–I)** As above, but reporting the simulated performance of Betasort. Reproduced with permission (DOI: 10.6084/m9.figshare.7992032.v1). Copyright 2019, Jensen, Terrace, and Ferrera.

Figure 10 depicts model-based performance for this manipulation. Both RL-Elo and SMC have their performance disrupted in a manner almost identical to the disruption experienced by *Q*-learning and VTM, and for the same reason: all four algorithms only apply informative feedback to the items present in the current trial. SMC’s resistance to this disruption is mainly due to its high level of accuracy overall.

Betasort’s displayed a clear distance effect by the end of the first phase of training, but all pairs (other than those that include G) drop to chance levels during FG training. However, within 20 trials of all-pairs testing, the original order sprang back into place. This effect is due to the combination of three factors: The relatively low value of φ (which causes *U* and *L* to shrink rapidly, without ever equaling zero), the algorithm’s implicit updating (which preserves items in a particular order by updating all positions, even when U and L are small), and the Jeffrey’s prior that adds 0.5 to the values of *U* and *L* in Equation 6. In effect, Betasort has preserved the *relative* order through FG training, but has allowed the *absolute* distance between items to become small as erroneous responses to G gradually push the values of $\frac{U}{U+L}$ for all other stimuli up to nearly 1.0. As items are bunched up against the same axis, the influence of the prior grows stronger, tending performance toward chance levels. However, as soon as pairs other than FG are introduced, the stimuli push themselves back apart (all while preserving their relative order) and the prior’s influence returns to background levels.

### Reversal of Stimulus Order at Test

We ran simulations in which algorithms were first trained on all 21 stimulus pairs for 210 trials (5 blocks, counterbalanced for position). Then, the stimulus order was reversed, becoming GFEDCBA. This new ordering was presented for another 210 trials.

**FIGURE 11:**
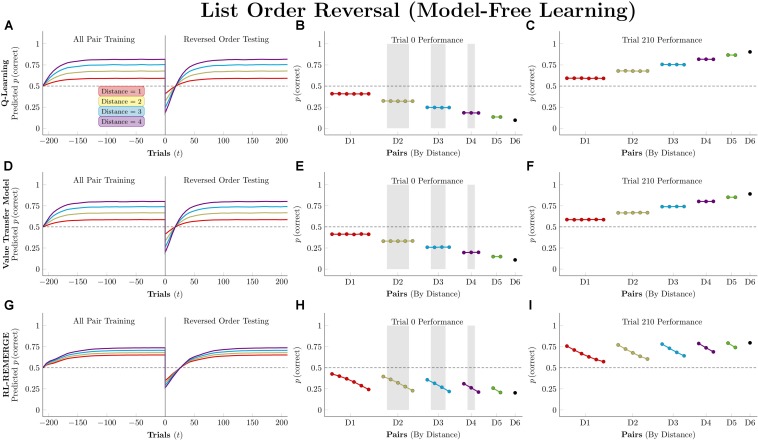
Performance of model-free algorithms on a 7-item pairwise serial learning task, presenting all 21 pairs during training and then reversing the order of the stimuli during testing. **(A)** Mean performance of *Q*-learning on non-terminal pairs throughout training and testing, sorted by symbolic distance. **(B)** Mean performance of *Q*-learning for each of the 21 pairs at transfer, sorted by symbolic distance. Critical pairs are shaded in gray. **(C)** Mean performance of *Q*-learning for each of the 21 pairs at the end of training. **(D–F)** As above, but reporting simulated performance of the Value Transfer Model (VTM). **(G–I)** As above, but reporting the simulated performance of RL-REMERGE. Reproduced with permission (DOI: 10.6084/m9.figshare.7992038.v1). Copyright 2019, Jensen, Terrace, and Ferrera.

Figure 11 depicts performance by the model-free algorithms. Because all pairs were being presented, all three successfully determined the ordering. Because the actors have been given no indication of the reversal at trial zero, their performance is a mirror image of their training performance, consistently below chance. By the end of testing, all three have recovered the overall pattern of accuracy seen during training, but they differ in terms of the number of trials needed to do so. *Q*-learning was the slowest of the three, with the order flipped following trial 27, on average. RL-REMERGE was only slightly faster, reversing its representation of the order after 26 trials. By this test, VTM was the nimblest, successfully reversing its order after 17 trials.

**FIGURE 12:**
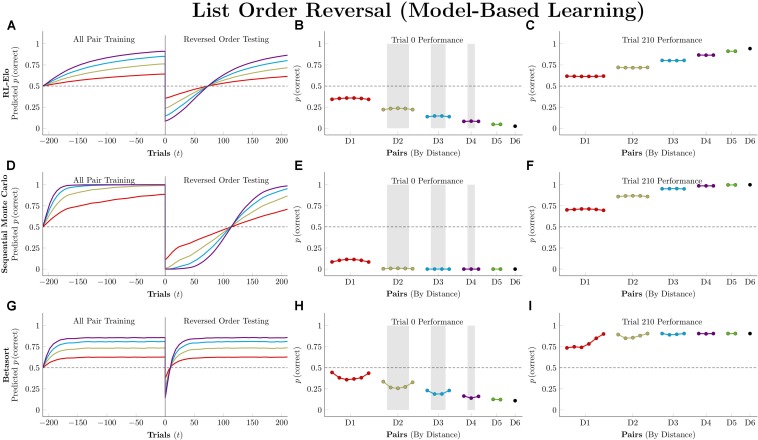
Performance of model-based algorithms on a 7-item pairwise serial learning task, presenting all 21 pairs during training and then reversing the order of the stimuli during testing. **(A)** Mean performance of RL-Elo on non-terminal pairs throughout training and testing, sorted by symbolic distance. **(B)** Mean performance of RL-Elo for each of the 21 pairs at transfer, sorted by symbolic distance. Critical pairs are shaded in gray. **(C)** Mean performance of RL-Elo for each of the 21 pairs at the end of training. **(D–F)** As above, but reporting simulated performance of the Sequential Monte Carlo (SMC) algorithm. **(G–I)** As above, but reporting the simulated performance of Betasort. Reproduced with permission (DOI: 10.6084/m9.figshare.7992041.v1). Copyright 2019, Jensen, Terrace, and Ferrera.

Figure 12 plots the effects of order reversal for the three model-based algorithms. Here, a much more dramatic disruption is seen. Although RL-Elo recovers its original level of performance, it takes an average of 74 trials to return the critical pairs to their correct order. SMC is even more disrupted – its near-ceiling performance translated to near-floor performance at the start of testing, and 210 trials was not sufficient training to recover its baseline performance, only managing to order the critical pairs correctly after 114 trials. In both cases, the culprit is a parameter (δ in the case of RL-Elo and σ in the case of SMC) that can only make small adjustments to their representations.

Contrastingly, the Betasort algorithm reorders the pairs correctly after just 9 trials, and recovers its original ceiling levels of accuracy by the end of the first block of testing. This owes to the low value of φ, which rapidly discards representation information that produces consistently incorrect responses.

### Linking Lists by Training a Single Stimulus Pair

One of the most dramatic series of results in the serial learning literature are the “list linking” studies reported by Treichler and colleagues. Monkeys learned the order of multiple separate lists using only adjacent pairs (e.g., ABCDE and FGHIJ). When monkeys were subsequently trained that E > F (in effect “linking” the end of one list to the start of the next), their performance at test was consistent with an inference that the two sublists should be combined into a single 10-item list, ABCDEFGHIJ (Treichler and Van Tilburg, 1996). List linking effects of this kind have since proved to be surprisingly robust over lists of fairly dramatic lengths, reaching well into the double digits (Treichler et al., 2007). However, if monkeys were not given training on the linking pair EF, their performance suggested that they assumed that item ranks were transferable to new pairings (Treichler and Raghanti, 2010). For example, after learning the lists ABCDE and FGHIJ, a monkey would be expected to favor G over D (because the former had a rank of 2 and the latter had a rank of 4) *unless* they were also trained that E > F, in which case the preference would reverse (because all items in the first list are implied by the EF pairing to have lower rank than any items in the second list).

The principle that rank should be transferable across lists is consistent with model-based accounts of serial learning, and is supported by a variety of experimental results (Chen et al., 1997; Merritt and Terrace, 2011; Kao et al., 2018). Insofar as each model makes inferences about position, then it stands to reason that those positions be comparable. However, no computational model of serial learning has yet been able to explain Treichler’s list linking results.

To simulate such an experiment, we trained the adjacent pairs of two five item lists (ABCDE and FGHIJ) for 17 blocks (4 pairs each, counterbalanced for position, yielding 16 trials per block, and 272 trials total). In the Linking condition, this training was followed by 34 trials of training only the pair EF. In the No Linking condition, these extra 34 trials of training were skipped. Finally, the actors were trained on all 45 pairs from the resulting 10-item linked list, ABCDEFGHIJ for 5 blocks (i.e., 450 trials, given stimulus counterbalancing).

**FIGURE 13:**
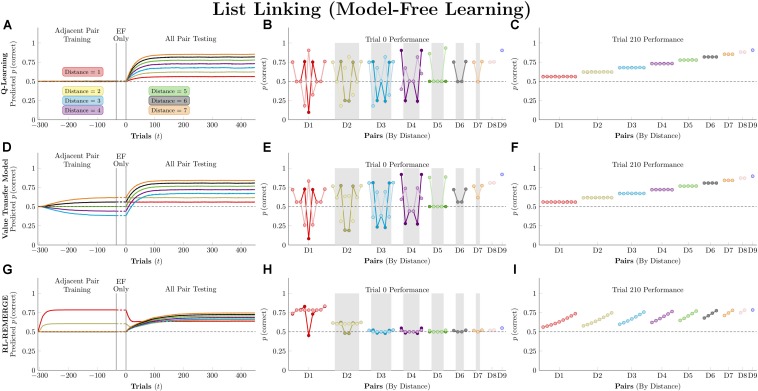
Performance of model-free algorithms on a 10-item list-linking procedure. During training, two 5-item lists were trained (adjacent pairs only). In the Linking condition, actors received training on the linking pair EF. In the No Linking condition, no extra training on EF was provided. Actors were then tested on the 45 possible pairings in the resulting 10-item list. **(A)** Mean performance of *Q*-learning on non-terminal pairs throughout training and testing, sorted by symbolic distance. After training, dashed lines represent the Linking condition, while solid lines represent the No Linking condition. **(B)** Mean performance of *Q*-learning for each of the 45 pairs at transfer, sorted by symbolic distance. Light-colored points represent the Linking condition, while dark-colored points represent the No Linking condition. Critical pairs are shaded in gray. **(C)** Mean performance of *Q*-learning for each of the 45 pairs at the end of training. **(D–F)** As above, but reporting simulated performance of the Value Transfer Model (VTM). **(G–I)** As above, but reporting the simulated performance of RL-REMERGE. Reproduced with permission (DOI: 10.6084/m9.figshare.7992044.v1). Copyright 2019, Jensen, Terrace, and Ferrera.

Figure 13 plots the effects of this simulated experiment. *Q*-learning’s overall response accuracy for critical pairs is strictly at chance levels, but performance for individual pairs was a mixture of trials that were either very low or very high in accuracy. EF training had the effect of flipping preference for some of these pairs, but these effects canceled out, leaving the Linking and No Linking conditions no different on average. Although this effect was also visible for VTM, the net effect was that performance on some critical pairs was above chance at the end of training, while other critical pairs were below chance. RL-REMERGE, by contrast, displayed almost no difference between the two conditions at any stage of training. The only substantive difference was that the pairs EF, DF, and EG were all above chance in the Linking condition. Although this is not a dramatic effect, it is the only result we found that yields consistently-above-chance predictions for a list linking manipulation.

**FIGURE 14:**
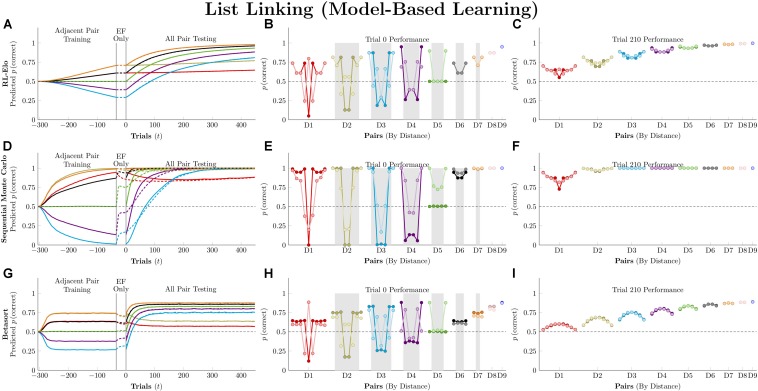
Performance of model-based algorithms on a 10-item list-linking procedure. During training, two 5-item lists were trained (adjacent pairs only). In the Linking condition, actors received training on the linking pair EF. In the No Linking condition, no extra training on EF was provided. Actors were then tested on the 45 possible pairings in the resulting 10-item list. **(A)** Mean performance of RL-Elo on non-terminal pairs throughout training and testing, sorted by symbolic distance. After training, dashed lines represent the Linking condition, while solid lines represent the No Linking condition. **(B)** Mean performance of RL-Elo for each of the 45 pairs at transfer, sorted by symbolic distance. Light-colored points represent the Linking condition, while dark-colored points represent the No Linking condition. Critical pairs are shaded in gray. **(C)** Mean performance of RL-Elo for each of the 45 pairs at the end of training. **(D–F)** As above, but reporting simulated performance of the Sequential Monte Carlo (SMC) algorithm. **(G–I)** As above, but reporting the simulated performance of Betasort. Reproduced with permission (DOI: 10.6084/m9.figshare.7992047.v1). Copyright 2019, Jensen, Terrace, and Ferrera.

Figure 14 plots the effects of list linking on the performance of the model-based algorithms. RL-Elo displays a pattern of behavior similar to that of VTM, performing better than chance on some pairs at test, but worse than chance on others. It experienced no benefit from the extra training on EF. Contrastingly, although SMC initially showed a similar pattern, it showed visible benefits from EF training, improving its performance for nearly all critical pairs. Although this was not enough to bring every critical pair above chance, it nevertheless constituted a substantial gain. We interpret this as being a consequence of its crowdsourcing: Since the relative positions of the two lists were allowed to drift with respect to one another, some of the 10,000 candidate orderings were closer to a full separation of the two lists, and these “partial solutions” received a big boost during EF training because they were the ones that tended to view E as having a lower position than F. Finally, Betasort’s performance during training was more in keeping with the patterns displayed by VTM and RL-Elo. Despite this, EF training had a visible effect, converging the critical pairs toward chance levels. However, this effect was not due to the algorithm converging on a better solution. It was instead the effect of the recall parameter φ relaxing the model for an additional 34 trials.

Although no algorithms solved the list linking problem, the two that came closest were RL-REMERGE (which learned some information from EF training and never displayed dramatic below-chance performance) and SMC (which appeared to stumble upon a successful subset of models among its current 10,000 candidate orderings).

## Model Comparison and Ensemble Modeling

Our emphasis thus far has been to qualitatively describe the performance of the algorithms, in order to call attention to the characteristics of behavior that arise consequent to each algorithm’s value updating function and action policy. Judging whether an algorithm provides the “best fit” is not merely a matter specifying a particular experimental condition, but also of identifying the particular learning epoch that is of experimental interest. Even if no single model gives a comprehensive account of observed behavior, identifying particular epochs that resemble the performance of one algorithm or another can potentially revealing underlying mechanisms.

**FIGURE 15:**
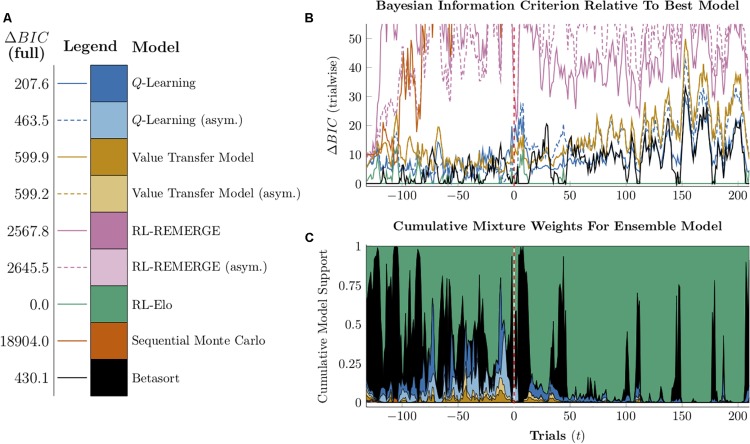
Model comparison of nine learning algorithms performing transitive inference. **(A)** Comparison for model-free algorithms (symmetric and asymmetric updating) and model-based algorithm using the full Hubble dataset, as well as figure legend for remaining panels. Δ*BIC* refers to the Bayesian information criterion computed for each model, minus the value computed for the best model. **(B)** Value of Δ*BIC* for each model, computed trialwise using a 3-trial moving window. Trial zero corresponds with the start of all-pairs testing, denoted by a red-and-white dashed line. **(C)** Cumulative plot of the weights for an ensemble model with contributions from all nine algorithms. Weights for Bayesian averaging were approximated using *exp*⁡(−0.5⋅Δ*BIC*_*i*_) for each algorithm *i*. Reproduced with permission (DOI: 10.6084/m9.figshare.8851700.v1). Copyright 2019, Jensen, Terrace, and Ferrera.

Using the full Hubble dataset as the empirical ground truth, a score was computed each algorithm using the Bayesian information criterion (*BIC*) (Schwarz, 1978). Since the absolute scale of BIC scores is arbitrary, we also computed Δ*BIC*, in which each score has the minimum observed score subtracted from its value. Since lower scores correspond with better fit, the best-fitting model overall receives a Δ*BIC* of 0.0. These scores are presented in Figure 15A. In this comprehensive scoring, the RL-Elo model provided the best fit by a substantial margin, followed by symmetric-updating *Q*-learning, and then by Betasort. Based on Figures 6, 7, it appears as though *Q*-learning beat out Betasort because, although Betasort displayed a symbolic distance effect at transfer, it did not display a terminal item effect. RL-Elo proved the best-fitting model because its behavior displayed both of these phenomena.

Figure 15B plots the values of Δ*BIC* for each algorithm based only on a three-trial moving window of data (153 trials drawn from Hubble’s 51 sessions). Δ*BIC* can in turn be used as a simple approximation of the weights used by an ensemble model, under the framework of Bayesian model averaging (Raftery, 1996), with each weight given by *exp*⁡(−0.5⋅Δ*BIC*) and the proportional support for each model obtained by dividing each weight by the sum of all weights. These measures of proportional support are plotted in Figure 15C as a cumulative plot, such that the relative contribution of each model is given by its vertical cross-section at trial *t*.

Figures 15B,C show that RL-Elo (in green) usually gave the best description of behavior at any given point during learning with an average contribution of 69.4% across all trials. Betasort provided a supporting role, averaging a contribution of 22.2% across all trials, while *Q*-learning displayed brief bursts of relevance (averaging a contribution of 3.8% for symmetric updating and 2.7% for asymmetric updating). However, despite RL-Elo generally being dominant in the ensemble, it was not the dominant model in all epochs of learning. During the early stages of learning, Betasort often gave a better description of behavior. Later in training, model-free algorithms based on associative strength (i.e., *Q*-learning and VTM) became more compelling candidates. Betasort was the preferred model during the first block of testing (i.e., trials 0–20, which included the first time each of the non-adjacent pairs was presented). For the remainder of testing however, RL-Elo dominated the ensemble model, with only occasional periods of advantage for Betasort. Throughout both training and testing, RL-REMERGE and SMC poorly described behavior, resulting in negligible contributions to the ensemble model.

Despite the ensemble model being overwhelmingly determined by the model-based algorithms, the period of relevance of model-free learning during the later stages of training could be seen as evidence of a shift from one style of learning to another. It is now widely held that organisms likely use multiple simultaneous systems for solving complex problem, but disagreement remains over whether these strategies work in parallel or cue one another in a flexible sequential fashion (Daw, 2012). The shift in the relevance of models over the course of training is suggestive of such a “two-step” approach.

It is worth emphasizing that although SMC fails utterly as a model of Hubble’s behavior, it does not fail to perform TIs. Figure 7 demonstrates unambiguously that SMC infers the ordering of the stimulus pairs, performs above chance on all stimulus pairs, and displays both the symbolic distance effect and the terminal item effect. Its abysmal Δ*BIC* scores (which mostly disappear off the top of the scale in Figure 15B) reflect performance that is much too good to be a plausible model of TI learning in monkeys. This is an example of a wider pattern in the machine learning literature, in which many algorithms that perform their assigned tasks too well are poor models for neural processes. Simulation and comparison to empirical data provides a way of falsifying such models (Palminteri et al., 2017).

By contrast, RL-Elo is a consistently compelling model because, in addition to its behavior possessing the same qualitative features as SMC, it also approximates the overall error rate observed in Hubble’s data. That said, it is also important to note that RL-Elo’s performance is disrupted by “massed trial” manipulations (as shown in Figure 10), which the empirical literature has found do not undermine serial order representations in animals.

## A Note on Computational Complexity

Several factors are considered when comparing the complexity of algorithms. The most straightforward are its memory demands: How many values must the algorithm keep track of at each stage of its operation? Another obvious measure is its operational speed: How long does the algorithm take to complete a series of decisions given reasonable inputs? Finally, it is common to discuss how an algorithm’s runtime scales as a function of its inputs: As the number of input items increases, how is runtime expected to grow?

By these criteria, four of the algorithms in this manuscript are clear winners: *Q*-learning, VTM, RL-Elo, and Betasort. The memory demands of each scales linearly with list length *n*: *Q*-learning, VTM, and RL-Elo each keep track of *n* values in memory, while Betasort keeps track of 4*n* values. They are also computationally rapid. We used a 2016 Macbook Pro to simulate 42,000 trials of a TI task using all pairs from a 7-item list, and this took 0.96 s for *Q*-learning, 1.26 s for VTM, 1.25 s for RL-Elo, and 1.68 s for Betasort. Finally, all four algorithms are expected have order *n* complexity, scaling their runtime chiefly as a function of the list length.

RL-REMERGE and SMC perform comparatively poorly. In the case of RL-REMERGE, the Conjunctive layer ensures that its memory and runtime demand grow on the order of *n*^2^. Even at reasonable list lengths, its iterative internal use of renormalization to discover a steady state is computationally intensive (42,000 simulated trials took 2879.46 s). While SMC scales more reasonably, with growth of order *n*, its 10,000 hypotheses constitute a substantial opportunity cost, both in terms of memory and processing. These are reflected in its costly runtime: 42,000 simulated trials took SMC 653.72 s.

However, these measures are somewhat unfair to both because these algorithms represent distributed solutions to the TI problem. Renormalization of neural networks, for example, is merely a mathematical trick to emulate the behavior of a distributed physical system, so RL-REMERGE’s *n*^2^ growth is mainly a problem of memory. Provided one is willing to allocate the neurons needed, processing time would be distributed accordingly. In the same vein, SMC can be understood as 10,000 parallel implementations of RL-Elo. Provided one is willing to distribute the problem in this way (and make use of many processor cores to do so), SMC’s performance should be very similar to RL-Elo (although the computational burden of periodic resampling of the list orders is less easily resolved).

Our view is that although complexity should be taken seriously when comparing computational models, it should also be recognized that these algorithms are primarily high-level descriptions of behavior. The Betasort algorithm should not, for example, be viewed as a proposal that organisms are actually computing and drawing random samples from beta distributions. Rather, the beta distribution is the maximum entropy distribution for the parameter uncertainty of Bernoulli processes, and as such there are many physical systems for which it will provide a good approximation. Any of the algorithms in this manuscript could be seen as a higher-level description of some distributed process, and RL-REMERGE and SMC appear less efficient because they explicitly distribute their processing.

With these considerations in mind, we consider RL-REMERGE to be the least plausible of the algorithms, since it requires implementing nodes for every “context.” In standard TI experiments, this takes the form of stimulus pairs, but it is easy to imagine a task in which subjects have to choose from among three or four stimuli, causing the Conjunctive layers to grow even faster since it would need a node for every stimulus combination. Contrastingly, the other five algorithms are all able to scale linearly as a function of list length, regardless of the number of stimuli presented in any given trial.

## Conclusion

The algorithms in this manuscript represent a cross-section of different computational methods for explaining TI performance. These methods succeed to varying degrees in discovering the structure of ordered sets, even when stimuli are presented pairwise without explicit spatial or temporal cues. Our goal was to gain insight about how each handles challenges that arise in natural settings, such as unequal rewards and base rates of presentation, as well as established experimental manipulation, such as list linking. In every case other than *Q*-learning (which was included to provide a model-free baseline), an argument can be made that each of these algorithms can, to some extent, “solve” TI problems. The patterns of error vary dramatically from one algorithm to the next, however, as do the effects of variations in the procedure.

While we can still learn a great deal from traditional TI procedures, the ability to implement theories of TI as computational models demands a shift in the experimental approach used to study serial learning. In the past, experimentalists have largely avoided making dramatic changes to experimental procedures because their theories were not sufficiently clear about what behavior would be expected under these new designs. By making computational simulation a part of both theorizing and of experimental design, future experiments can much more rapidly identify areas of discrepancy between theory and behavior, which in turn lead to both stronger theories and to more informative experiments.

## Author Contributions

GJ performed the statistical analysis and wrote the first draft of the manuscript. GJ and VF contributed code to the study. All authors contributed to conception and design of the study, manuscript revision, and read and approved the submitted version.

## Conflict of Interest Statement

The authors declare that the research was conducted in the absence of any commercial or financial relationships that could be construed as a potential conflict of interest.
